# Supplementary material for: Risk Stratification for Management of Solitary Fibrous Tumor/Hemangiopericytoma of the Central Nervous System
Source: Cancers (Basel). 2023 Jan 31;15(3):876. doi: 10.3390/cancers15030876 (PMC9913704; doi:10.3390/cancers15030876)
Supplement: Supplementary file 1 [file cancers-15-00876-s001.zip › Supplemental Figures S1 and S2.pdf]

A

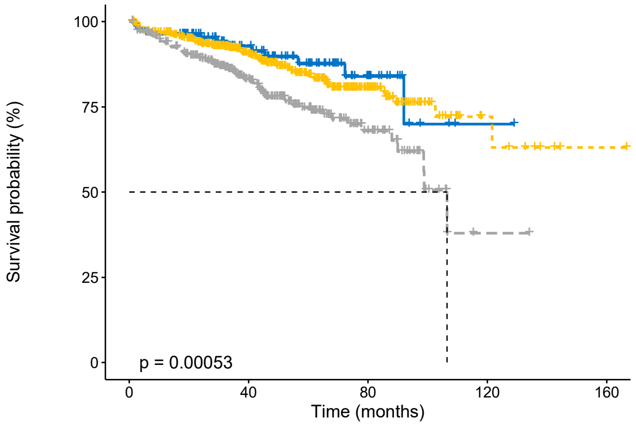

Number at risk

	0	40	80	120	160
Grade=G1	111	63	17	1	0
Grade=G2	428	259	69	8	1
Grade=G3	303	159	34	1	0

Strata — Grade=G1 — Grade=G2 — Grade=G3

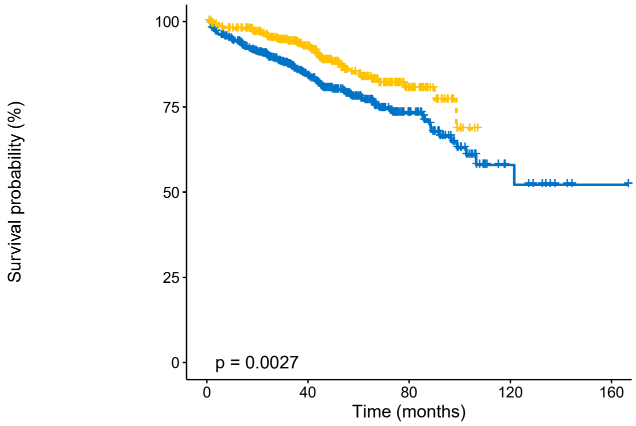

Number at risk

	0	40	80	120	160
EOR=No surgery/STR	474	260	80	10	1
EOR=GTR	392	234	48	0	0

Strata — EOR=No surgery/STR — EOR=GTR

B

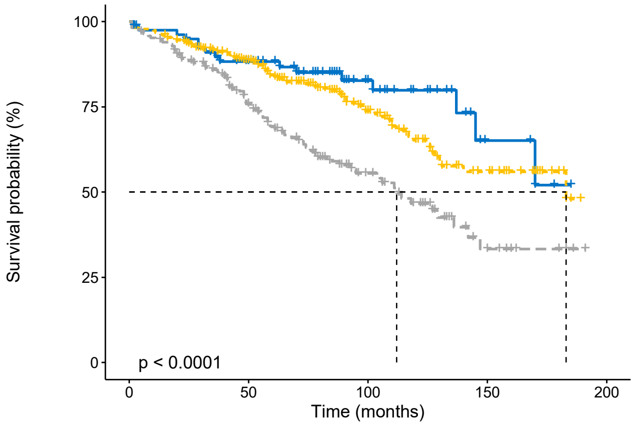

Number at risk

	0	50	100	150	200
Grade=G1	80	61	30	6	0
Grade=G2	275	197	92	29	0
Grade=G3	167	99	41	10	0

Strata — Grade=G1 — Grade=G2 — Grade=G3

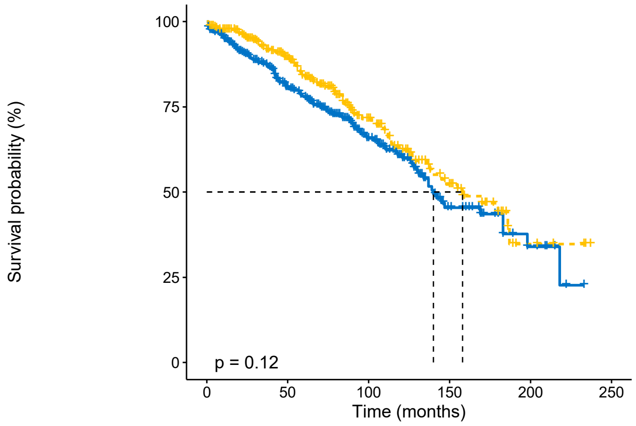

Number at risk

	0	50	100	150	200	250
EOR=No surgery/STR	350	206	104	31	7	0
EOR=GTR	296	194	90	36	5	0

Strata — EOR=No surgery/STR — EOR=GTR

C

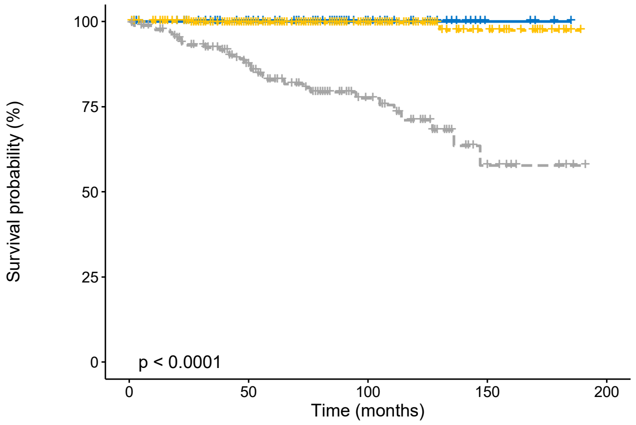

Number at risk

	0	50	100	150	200
Grade=G1	80	61	30	6	0
Grade=G2	275	197	92	29	0
Grade=G3	165	98	40	10	0

Strata — Grade=G1 — Grade=G2 — Grade=G3

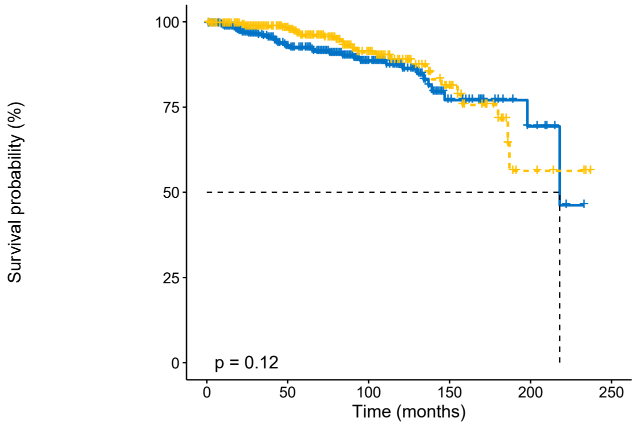

Number at risk

	0	50	100	150	200	250
EOR=No surgery/STR	349	205	103	31	7	0
EOR=GTR	295	194	90	36	5	0

Strata — EOR=No surgery/STR — EOR=GTR

Supplemental Figure S1- Kaplan-Meier curves for overall survival in the NCDB (A) and overall (B) and cause-specific survival (C) in the SEER database based on grade and EOR.

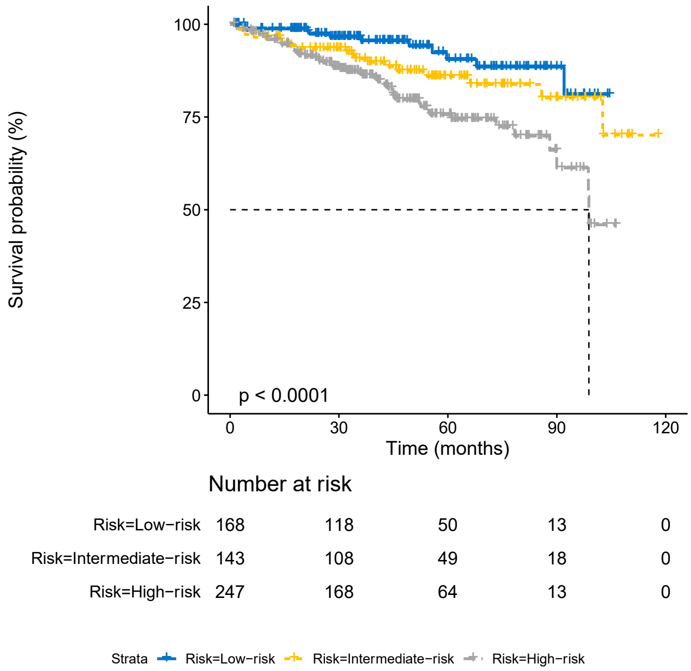

Supplemental Figure S2- Sensitivity Analysis: Kaplan-Meier curves for overall survival in the NCDB, including patients only with all histological datapoints.
