# Supplementary material for: Risk Stratification for Management of Solitary Fibrous Tumor/Hemangiopericytoma of the Central Nervous System
Source: Cancers (Basel). 2023 Jan 31;15(3):876. doi: 10.3390/cancers15030876 (PMC9913704; doi:10.3390/cancers15030876)
Supplement: Supplementary file 1 [file cancers-15-00876-s001.zip › Supplemental Table S1.pdf]

Supplemental Table S1- Demographic and Clinical Characteristics of the SEER Cohort

Characteristic	Low-risk, N = 204[1]	Intermediate-risk, N = 151[1]	High-risk, N = 167[1]
Age	54 (43, 65)	54 (42, 64)	55 (44, 65)
Sex			
Female	97 (48%)	77 (51%)	93 (56%)
Male	107 (52%)	74 (49%)	74 (44%)
Race			
White	162 (91%)	130 (95%)	133 (88%)
Asian or Pacific Islander	0 (0%)	0 (0%)	0 (0%)
Black	16 (8.9%)	7 (5.1%)	17 (11%)
American Indian/Alaska Native	0 (0%)	0 (0%)	0 (0%)
Unknown	1 (0.6%)	0 (0%)	1 (0.7%)
Grade			
G1	80 (39%)	0 (0%)	0 (0%)
G2	124 (61%)	151 (100%)	0 (0%)
G3	0 (0%)	0 (0%)	167 (100%)
Site			
Brain	150 (74%)	109 (72%)	156 (93%)
Spinal/Other CNS	54 (26%)	42 (28%)	11 (6.6%)
EOR			
No surgery/STR	49 (24%)	151 (100%)	85 (51%)
GTR	155 (76%)	0 (0%)	82 (49%)
Vital Status			
Alive	165 (81%)	102 (68%)	97 (58%)
Dead	39 (19%)	49 (32%)	70 (42%)
Cause- Specific Death			
Alive or died of other cause	204 (100%)	149 (99%)	132 (80%)
Died because of this tumor	0 (0%)	2 (1.3%)	33 (20%)
Unknown	0	0	2
Follow-Up Time	80 (51, 118)	74 (42, 110)	58 (38, 99)

[1]Median (IQR); n (%)
