# Supplementary material for: Risk Stratification for Management of Solitary Fibrous Tumor/Hemangiopericytoma of the Central Nervous System
Source: Cancers (Basel). 2023 Jan 31;15(3):876. doi: 10.3390/cancers15030876 (PMC9913704; doi:10.3390/cancers15030876)
Supplement: Supplementary file 1 [file cancers-15-00876-s001.zip › Supplemental Table S10.pdf]

| | Univariable | | | Multivariable | | |
|---|---|---|---|---|---|---|
| Characteristic | HR[1] | 95% CI[1] | p-value | HR[1] | 95% CI[1] | p-value |
| **Age** | 1.07 | 1.04, 1.09 | **<0.001** | 1.07 | 1.04, 1.09 | **<0.001** |
| **Sex** | | | | | | |
| Male | — | — | | | | |
| Female | 0.85 | 0.44, 1.66 | 0.64 | | | |
| **Race** | | | | | | |
| White | — | — | | | | |
| Black | 0.76 | 0.23, 2.48 | 0.64 | | | |
| Other/Unknown | 0.00 | 0.00, Inf | >0.99 | | | |
| Asian/Pacific Islander | 0.00 | 0.00, Inf | >0.99 | | | |
| **Charlson-Deyo Comorbidity Index** | | | | | | |
| 0 | — | — | | | | |
| 1 | 1.62 | 0.73, 3.59 | 0.23 | | | |
| 2 or more | 0.45 | 0.06, 3.29 | 0.43 | | | |
| **Site** | | | | | | |
| Brain | — | — | | | | |
| Spinal/Other CNS | 0.49 | 0.19, 1.26 | 0.14 | | | |
| **Tumor Size** | | | | | | |
| 5cm or less | — | — | | | | |
| Greater than 5cm | 1.08 | 0.50, 2.37 | 0.84 | | | |
| Unknown | 0.62 | 0.27, 1.45 | 0.27 | | | |
| **Radiation** | | | | | | |
| No radiotherapy | — | — | | | | |
| Radiotherapy | 0.65 | 0.32, 1.31 | 0.23 | | | |

[1]HR = Hazard Ratio, CI = Confidence Interval

Supplemental Table S10- Univariable and Multivariable Analysis of Overall Survival in the Intermediate-Risk Group at 6-Month Landmark
