# Supplementary material for: Risk Stratification for Management of Solitary Fibrous Tumor/Hemangiopericytoma of the Central Nervous System
Source: Cancers (Basel). 2023 Jan 31;15(3):876. doi: 10.3390/cancers15030876 (PMC9913704; doi:10.3390/cancers15030876)
Supplement: Supplementary file 1 [file cancers-15-00876-s001.zip › Supplemental Table S2.pdf]

	Univariable			Multivariable		
Characteristic	HR[1]	95% CI[1]	p-value	HR[1]	95% CI[1]	p-value
**Age**	1.06	1.05, 1.08	**<0.001**	1.06	1.05, 1.08	**<0.001**
**Sex**						
Male	—	—				
Female	0.77	0.55, 1.06	0.10			
**Race**						
White	—	—				
Black	0.58	0.30, 1.14	0.11			
Other/Unknown	0.50	0.12, 2.04	0.34			
Asian/Pacific Islander	0.33	0.11, 1.05	0.061			
**Charlson-Deyo Comorbidity Index**						
0	—	—		—	—	
1	1.29	0.84, 1.97	0.24	0.92	0.60, 1.41	0.71
2 or more	1.80	1.08, 3.01	**0.025**	1.47	0.88, 2.47	0.14
**Tumor Size**						
5cm or less	—	—				
Greater than 5cm	0.90	0.61, 1.32	0.58			
Unknown	1.10	0.74, 1.62	0.64			
**Site**						
Brain	—	—				
Spinal/Other CNS	0.88	0.59, 1.32	0.54			
**Risk**						
Low-risk	—	—		—	—	
Intermediate-risk	1.60	1.01, 2.55	**0.045**	1.52	0.95, 2.41	0.079
High-risk	2.56	1.68, 3.89	**<0.001**	2.38	1.56, 3.63	**<0.001**
**Radiation**						
No radiotherapy	—	—				
Radiotherapy	0.84	0.61, 1.17	0.30			

	Univariable			Multivariable		
Characteristic	HR[1]	95% CI[1]	p-value	HR[1]	95% CI[1]	p-value

[1]HR = Hazard Ratio, CI = Confidence Interval

Supplemental Table S2- Univariable and Multivariable Analysis of Overall Survival in the NCDB
