# Supplementary material for: Risk Stratification for Management of Solitary Fibrous Tumor/Hemangiopericytoma of the Central Nervous System
Source: Cancers (Basel). 2023 Jan 31;15(3):876. doi: 10.3390/cancers15030876 (PMC9913704; doi:10.3390/cancers15030876)
Supplement: Supplementary file 1 [file cancers-15-00876-s001.zip › Supplemental Table S3.pdf]

	Univariable			Multivariable		
Characteristic	HR[1]	95% CI[1]	p-value	HR[1]	95% CI[1]	p-value
**Age**	1.04	1.03, 1.05	**<0.001**	1.04	1.03, 1.05	**<0.001**
**Sex**						
Female	—	—				
Male	1.01	0.76, 1.33	0.96			
**Race**						
White	—	—				
Asian or Pacific Islander						
Black	1.02	0.61, 1.70	0.94			
American Indian/Alaska Native						
Unknown	0.57	0.08, 4.06	0.57			
**Site**						
Brain	—	—		—	—	
Spinal/Other CNS	0.59	0.38, 0.92	**0.020**	0.66	0.41, 1.07	0.090
**Risk**						
Low-risk	—	—		—	—	
Intermediate-risk	1.90	1.25, 2.90	**0.003**	1.94	1.27, 2.95	**0.002**
High-risk	2.76	1.86, 4.08	**<0.001**	2.62	1.76, 3.92	**<0.001**

	Univariable			Multivariable		
Characteristic	HR[1]	95% CI[1]	p-value	HR[1]	95% CI[1]	p-value

[1]HR = Hazard Ratio, CI = Confidence Interval

Supplemental Table S3- Univariable and Multivariable Analysis of Overall Survival in SEER
