# Supplementary material for: Risk Stratification for Management of Solitary Fibrous Tumor/Hemangiopericytoma of the Central Nervous System
Source: Cancers (Basel). 2023 Jan 31;15(3):876. doi: 10.3390/cancers15030876 (PMC9913704; doi:10.3390/cancers15030876)
Supplement: Supplementary file 1 [file cancers-15-00876-s001.zip › Supplemental Table S4.pdf]

	Univariable			Multivariable		
Characteristic	HR[1]	95% CI[1]	p-value	HR[1]	95% CI[1]	p-value
**Age**	1.08	1.05, 1.11	**<0.001**	1.08	1.05, 1.11	**<0.001**
**Sex**						
Male	—	—				
Female	0.91	0.45, 1.84	0.79			
**Race**						
White	—	—				
Black	0.69	0.16, 2.90	0.61			
Other/Unknown	0.74	0.10, 5.43	0.76			
Asian/Pacific Islander	0.37	0.05, 2.71	0.33			
**Charlson-Deyo Comordbity Index**						
0	—	—		—	—	
1	0.85	0.29, 2.46	0.76	0.64	0.22, 1.86	0.41
2 or more	3.83	1.45, 10.1	**0.007**	3.49	1.30, 9.34	**0.013**
**Tumor Size**						
5cm or less	—	—				
Greater than 5cm	1.15	0.50, 2.65	0.75			
Unknown	1.94	0.80, 4.71	0.14			
**Site**						
Brain	—	—				
Spinal/Other CNS	1.00	0.43, 2.33	>0.99			
**Radiation**						
No radiotheray	—	—				
Radiotherapy	1.26	0.60, 2.65	0.55			

[1]HR = Hazard Ratio, CI = Confidence Interval

Supplemental Table S4- Univariable and Multivariable Analysis of Overall Survival in the Low-Risk Group.
