# Supplementary material for: Risk Stratification for Management of Solitary Fibrous Tumor/Hemangiopericytoma of the Central Nervous System
Source: Cancers (Basel). 2023 Jan 31;15(3):876. doi: 10.3390/cancers15030876 (PMC9913704; doi:10.3390/cancers15030876)
Supplement: Supplementary file 1 [file cancers-15-00876-s001.zip › Supplemental Table S5.pdf]

	Univariable			Multivariable		
Characteristic	HR[1]	95% CI[1]	p-value	HR[1]	95% CI[1]	p-value
**Age**	1.07	1.04, 1.09	**<0.001**	1.06	1.04, 1.09	**<0.001**
**Sex**						
Male	—	—				
Female	0.82	0.45, 1.49	0.52			
**Race**						
White	—	—				
Black	0.57	0.18, 1.85	0.35			
Other/Unknown	0.00	0.00, Inf	>0.99			
Asian/Pacific Islander	0.00	0.00, Inf	>0.99			
**Charlson-Deyo Comorbidity Index**						
0	—	—				
1	1.44	0.69, 3.01	0.33			
2 or more	0.70	0.17, 2.90	0.62			
**Tumor Size**						
5cm or Less	—	—				
Greater than 5cm	0.81	0.38, 1.70	0.58			
Unknown	0.62	0.30, 1.27	0.19			
**Site**						
Brain	—	—				
Spinal/Other CNS	0.83	0.41, 1.68	0.60			
**Radiation**						
No radiotherapy	—	—		—	—	
Radiotherapy	0.52	0.27, 0.99	**0.048**	0.74	0.38, 1.47	0.39

[1]HR = Hazard Ratio, CI = Confidence Interval

Supplemental Table S5- Univariable and Multivariable Analysis of Overall Survival in the Intermediate-Risk Group.
