# Supplementary material for: Risk Stratification for Management of Solitary Fibrous Tumor/Hemangiopericytoma of the Central Nervous System
Source: Cancers (Basel). 2023 Jan 31;15(3):876. doi: 10.3390/cancers15030876 (PMC9913704; doi:10.3390/cancers15030876)
Supplement: Supplementary file 1 [file cancers-15-00876-s001.zip › Supplemental Table S6.pdf]

Supplemental Table S6- Univariable and Multivariable Analysis of Overall Survival in the High-Risk Group.

	Univariable			Multivariable		
Characteristic	HR[1]	95% CI[1]	p-value	HR[1]	95% CI[1]	p-value
**Age**	1.05	1.04, 1.07	**<0.001**	1.05	1.03, 1.07	**<0.001**
**Sex**						
Male	—	—				
Female	0.70	0.44, 1.11	0.13			
**Race**						
White	—	—				
Black	0.56	0.20, 1.54	0.26			
Other/Unknown	0.56	0.08, 4.06	0.57			
Asian/Pacific Islander	0.57	0.14, 2.34	0.44			
**Charlson-Deyo Comorbidity Index**						
0	—	—				
1	1.41	0.78, 2.57	0.26			
2 or more	1.55	0.78, 3.06	0.21			
**Site**						
Brain	—	—				
Spinal/Other CNS	0.97	0.52, 1.82	0.94			
**Tumor Size**						
Less5cm	—	—				
Great5cm	0.95	0.54, 1.67	0.87			
Unknown	1.26	0.72, 2.18	0.42			
**EOR**						
No surgery/STR	—	—		—	—	
GTR	0.47	0.28, 0.76	**0.002**	0.54	0.33, 0.88	**0.014**
**Radiation**						
No radiotherapy	—	—		—	—	
Radiotherapy	0.46	0.29, 0.74	**0.001**	0.59	0.36, 0.95	**0.031**

[1]HR = Hazard Ratio, CI = Confidence Interval
