# Supplementary material for: Risk Stratification for Management of Solitary Fibrous Tumor/Hemangiopericytoma of the Central Nervous System
Source: Cancers (Basel). 2023 Jan 31;15(3):876. doi: 10.3390/cancers15030876 (PMC9913704; doi:10.3390/cancers15030876)
Supplement: Supplementary file 1 [file cancers-15-00876-s001.zip › Supplemental Table S7.pdf]

Supplemental Table S7- Univariable and Multivariable Analysis of Overall Survival in the High-Risk Group at 3-Month Landmark.

	Univariable			Multivariable		
Characteristic	HR[1]	95% CI[1]	p-value	HR[1]	95% CI[1]	p-value
**Age**	1.05	1.03, 1.07	**<0.001**	1.05	1.03, 1.07	**<0.001**
**Sex**						
Male	—	—		—	—	
Female	0.60	0.37, 0.98	**0.040**	0.55	0.33, 0.92	**0.022**
**Race**						
White	—	—				
Black	0.61	0.22, 1.68	0.34			
Other/Unknown	0.61	0.08, 4.41	0.62			
Asian/Pacific Islander	0.64	0.16, 2.62	0.53			
**Charlson-Deyo Comorbidity Index**						
0	—	—				
1	1.46	0.78, 2.72	0.23			
2 or more	1.73	0.87, 3.44	0.12			
**Site**						
Brain	—	—				
Spinal/Other CNS	0.85	0.43, 1.68	0.65			
**Tumor Size**						
5cm or less	—	—				
Greater than 5cm	0.85	0.47, 1.53	0.58			
Unknown	1.23	0.70, 2.18	0.47			
**EOR**						
No surgery/STR	—	—		—	—	
GTR	0.53	0.32, 0.87	**0.012**	0.69	0.41, 1.15	0.16
**Radiation**						
No radiotherapy	—	—		—	—	
Radiotherapy	0.51	0.31, 0.84	**0.008**	0.64	0.39, 1.07	0.087

[1]HR = Hazard Ratio, CI = Confidence Interval
