# Supplementary material for: Risk Stratification for Management of Solitary Fibrous Tumor/Hemangiopericytoma of the Central Nervous System
Source: Cancers (Basel). 2023 Jan 31;15(3):876. doi: 10.3390/cancers15030876 (PMC9913704; doi:10.3390/cancers15030876)
Supplement: Supplementary file 1 [file cancers-15-00876-s001.zip › Supplemental Table S8.pdf]

Supplemental Table S8- Univariable and Multivariable Analysis of Overall Survival in the High-Risk Group at 6-Month Landmark.

	Univariable			Multivariable		
Characteristic	HR[1]	95% CI[1]	p-value	HR[1]	95% CI[1]	p-value
**Age**	1.05	1.03, 1.07	**<0.001**	1.05	1.03, 1.07	**<0.001**
**Sex**						
Male	—	—				
Female	0.65	0.39, 1.06	0.082			
**Race**						
White	—	—				
Black	0.64	0.23, 1.76	0.38			
Other/Unknown	0.64	0.09, 4.61	0.65			
Asian/Pacific Islander	0.68	0.17, 2.78	0.59			
**Charlson-Deyo Comorbidity Index**						
0	—	—				
1	1.26	0.65, 2.45	0.50			
2 or more	1.78	0.89, 3.55	0.10			
**Site**						
Brain	—	—				
Spinal/Other CNS	0.89	0.45, 1.77	0.75			
**Tumor Size**						
5cm or less	—	—				
Greater than 5cm	0.83	0.45, 1.52	0.54			
Unknown	1.24	0.69, 2.23	0.47			
**EOR**						
No surgery/STR	—	—		—	—	
GTR	0.49	0.29, 0.83	**0.007**	0.55	0.33, 0.92	**0.024**
**Radiation**						
No radiotherapy	—	—		—	—	
Radiotherapy	0.54	0.32, 0.90	**0.017**	0.66	0.39, 1.11	0.11

[1]HR = Hazard Ratio, CI = Confidence Interval
