# Supplementary material for: Risk Stratification for Management of Solitary Fibrous Tumor/Hemangiopericytoma of the Central Nervous System
Source: Cancers (Basel). 2023 Jan 31;15(3):876. doi: 10.3390/cancers15030876 (PMC9913704; doi:10.3390/cancers15030876)
Supplement: Supplementary file 1 [file cancers-15-00876-s001.zip › Supplemental Table S9.pdf]

	Univariable			Multivariable		
Characteristic	HR[1]	95% CI[1]	p-value	HR[1]	95% CI[1]	p-value
**Age**	1.06	1.04, 1.09	**<0.001**	1.06	1.04, 1.09	**<0.001**
**Sex**						
Male	—	—				
Female	0.78	0.42, 1.44	0.42			
**Race**						
White	—	—				
Black	0.62	0.19, 2.02	0.43			
Other/Unknown	0.00	0.00, Inf	>0.99			
Asian/Pacific Islander	0.00	0.00, Inf	>0.99			
**Charlson-Deyo Comorbidity Index**						
0	—	—				
1	1.58	0.75, 3.34	0.23			
2 or more	0.77	0.18, 3.22	0.72			
**Site**						
Brain	—	—				
Spinal/Other CNS	0.80	0.38, 1.68	0.56			
**Tumor Size**						
5cm or less	—	—				
Greater than 5cm	0.93	0.43, 1.98	0.84			
Unknown	0.72	0.34, 1.50	0.38			
**Radiation**						
No radiotherapy	—	—				
Radiotherapy	0.57	0.29, 1.11	0.10			

[1]HR = Hazard Ratio, CI = Confidence Interval

Supplemental Table S9- Univariable and Multivariable Analysis of Overall Survival in the Intermediate-Risk Group at 3-Month Landmark
